# Chaperones and the Proteasome System: Regulating the Construction and Demolition of Striated Muscle

**DOI:** 10.3390/ijms19010032

**Published:** 2017-12-22

**Authors:** Casey Carlisle, Kendal Prill, Dave Pilgrim

**Affiliations:** Department of Biological Sciences, University of Alberta, Edmonton, AB T6G 2E9, Canada; carlisle@ualberta.ca (C.C.); kprill@ualberta.ca (K.P.)

**Keywords:** molecular chaperone, misfolded protein, protein degradation, protein complex assembly, homeostasis, HSP, sarcomere

## Abstract

Protein folding factors (chaperones) are required for many diverse cellular functions. In striated muscle, chaperones are required for contractile protein function, as well as the larger scale assembly of the basic unit of muscle, the sarcomere. The sarcomere is complex and composed of hundreds of proteins and the number of proteins and processes recognized to be regulated by chaperones has increased dramatically over the past decade. Research in the past ten years has begun to discover and characterize the chaperones involved in the assembly of the sarcomere at a rapid rate. Because of the dynamic nature of muscle, wear and tear damage is inevitable. Several systems, including chaperones and the ubiquitin proteasome system (UPS), have evolved to regulate protein turnover. Much of our knowledge of muscle development focuses on the formation of the sarcomere but recent work has begun to elucidate the requirement and role of chaperones and the UPS in sarcomere maintenance and disease. This review will cover the roles of chaperones in sarcomere assembly, the importance of chaperone homeostasis and the cooperation of chaperones and the UPS in sarcomere integrity and disease.

## 1. Introduction

The unique tertiary and quaternary structure of a protein is critical for its function. Under the right conditions of pH, temperature, solute concentration, intracellular ions and solvent, many proteins are capable of spontaneously folding due to the intramolecular forces of the amino acids of the linear protein chain [1,2,3,4]. However, the majority of proteins require assistance to achieve their tertiary structure, with estimates of 30% of newly synthesized proteins being targeted for degradation due to improper folding [5]. These numbers increase when taking into account proteins that need to be refolded in the event of damage or denaturation. Molecular chaperones aid in the folding of thousands of proteins by binding the hydrophobic amino acids of client proteins and preventing aggregates in a process that we have recognized for several decades [6,7,8,9].

Proteins can become damaged in a variety of ways, from changes in the environmental conditions (temperature, oxidative stress & pH, salts, intracellular ions), wear and tear damage over time, or incomplete folding due to an absence of necessary chaperones [3,4]. Once proteins become misfolded, aggregation may occur due to the exposure of hydrophobic amino acid residues. At its most severe state, these aggregates can overwhelm cellular processes and machinery such that the cell undergoes apoptosis [10,11,12]. Many diseases arise from the aggregation of cellular proteins caused by their misfolding, the most well studied being neurodegenerative diseases including Alzheimer’s, Parkinson’s, Huntington’s, as well as prion diseases [13,14]. However, many muscle diseases (myopathies; see Table 1) are associated with protein aggregation, including inclusion body myopathy [15] and protein aggregate myopathies (PAM) [16]. While the cooperation of chaperones and the proteasome system have been well studied in the neurodegenerative diseases listed above, the resulting models and concepts discovered in these systems have not been applied to striated muscle despite the abundance of protein aggregation related myopathies listed in Table 1. Therefore, one of the purposes of this review is to highlight our current knowledge of the roles of chaperone and UPS related responses in striated muscle with respect to what has been studied in other organ systems.

As the diversity of cell and tissue types increases in complex organisms, novel contexts created the need to combat aggregation of misfolded or damaged proteins increase accordingly. In dynamic tissues, a new layer of complexity is added to this process as protein homeostasis (proteostasis) must occur as the tissue moves. Striated muscle exemplifies this complexity, as hundreds of components must be properly synthesized, folded and incorporated into its basic contractile unit, the sarcomere, for the tissue to function.

**Figure 1:**
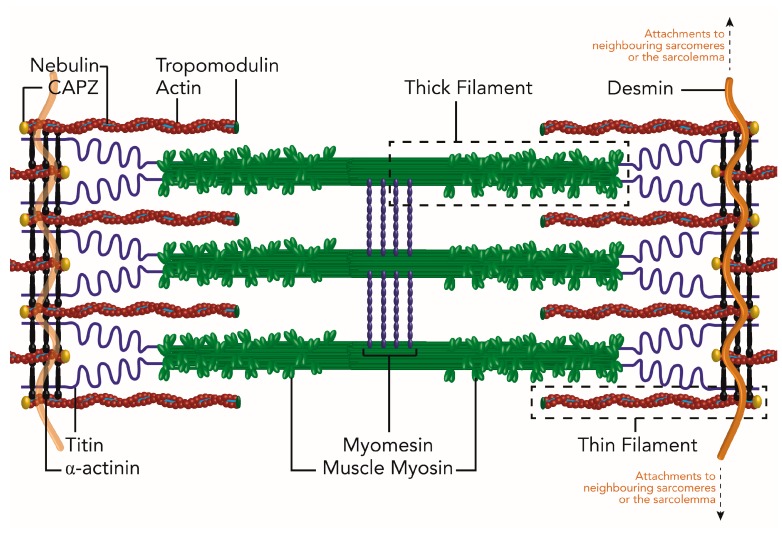
Simplified schematic of the mature sarcomere in striated muscle. The major components of the mature sarcomere are shown. The Z-disc, composed mainly of α-actinin, denote the boundaries of individual sarcomeres, add stability and act as attachment sites for signaling factors, neighboring myofibrils and the myocyte cell membrane (sarcolemma). Thin filaments made of actin, nebulin and tropomodulin extend from the Z-disc to interact with the globular head domains of the myosin thick filaments that protrude out from the M-line toward the Z-disc. The binding of myosin to actin, of the thin filaments, allows the thick filaments to pull the Z-discs toward the center of the sarcomere resulting in contraction/shortening of the sarcomere length. The protein giant, titin and the elastic properties of myomesin in the M-line buffer the contraction of the sarcomere. Desmin is incorporated into the Z-discs of sarcomeres to help stabilize sarcomere structure, align sarcomeres in neighboring myofibrils and connect the contractile structures to the sarcolemma.

The sarcomere is composed of four main parts: The Z-disc, the I-band, the A-band and the M line (Figure 1; [35,36]). The Z disk forms the boundaries of the sarcomere and while it is composed mainly of α-actinin, it houses and anchors hundreds of proteins, which gives this structure a diversity of functions. For a thorough review of Z-disc components, their roles and their resulting diseases, see reviews [37,38]. The Z-disc anchors nebulin, desmin and titin, which lead to the initial belief that the Z-disc had a purely sarcomere stabilizing function. However, its physical connections to the sarcolemma and reservoir of chaperones, co-chaperones and E3 ligases have led to an expansion of Z-disc roles to include signaling and mechanosensing. The I band is composed of troponin, tropomyosin and actin thin filaments [35]. Actin is anchored at the Z-disc by alpha-actinin and assembled in to filaments (F-actin) along the protein nebulin. In an ATP and calcium dependent manner, actin binds and releases myosin thick filaments, the main component of the A-band, to undergo muscle contraction [39]. Besides myosin, the A-band is home to MyBP-C and MyBP-H, the former of which is implicated in regulating thick filament thickness [35]. Also implicated in thick filaments assembly and register is the giant protein titin, which stretches one half of the sarcomere from Z-disc to M-line. Considered to be the third filament system of the sarcomere, titin has many functions [40,41] and whose role in sarcomere assembly, maintenance and disease, is still being uncovered. The final component of the sarcomere, the M-line, resides in the center of two Z-discs and is proposed to regulate sarcomere stability and monitor sarcomere integrity [24,25]. Components of the M-line are still being uncovered but include various isoforms of myomesin, creatine kinase and the C terminus of titin.

After construction of the sarcomere, damage resulting from repeated contractions forces the tissue to undergo constant dynamic remodeling to remain healthy. In skeletal muscle, multiple systems have been implicated in protein quality control, including the muscle chaperone system [42], the autophagy-lysosome system [43] and auxiliary factors such as proteases and calpains [44] and the ubiquitin proteasome system (UPS). The UPS has been widely studied for decades as a mechanism by which damaged proteins are degraded [45,46,47]. In short, the UPS relies on three enzymes, E1, E2 and E3, which together activate and attach ubiquitin to an internal lysine residue on a damaged client protein. E1 enzymes activate ubiquitin, a highly conserved, 76 amino acid protein [48], in an ATP dependent process. Once activated, ubiquitin is transferred to an E2 ubiquitin carrier protein and then either independently or with the help of an E3 ubiquitin ligase protein or complex, ubiquitin is attached to a client protein. This process repeats until the client protein is poly-ubiquitinated. While only a few E1 enzymes exist, there are an abundance of E2 or E3 enzymes, as they dictate client protein specificity. It has been proposed that up to 5% of the eukaryotic genome encodes UPS related components, with the human genome containing genes for approximately 600 different E3 enzymes [49]. Once ubiquitin tagged, the client protein is degraded by the proteasome, an enzyme composed of a 20S catalytic core and one 19S regulatory cap, forming the 26S proteasome, or two 19S regulatory caps, forming the 30S proteasome [50,51] UPS mediated degradation occurs through the 26S proteasome in striated muscle. The 19S regulatory cap is responsible for unwinding the protein, where it is fed into the 20S core and degraded. Ubiquitin, however, is removed by de-ubiquitinating enzymes and recycled for further use. It is important to note that ubiquitin tagging is reversible [52] and involved in processes outside of proteasome degradation. Furthermore, other ubiquitin-independent mechanisms exist of labelling damaged proteins for UPS degradation, as exemplified by the N-end rule [53,54]. Together, this indicates multiple pathways of protein turnover [55].

While proteases, calpains and autophagy are vastly important to understanding muscle turnover and proteostasis, in this review we choose to focus on the muscle chaperone system and its cooperation with the UPS as their communication has been described for many neurological systems but largely ignored in striated muscle. Therefore, we will discuss known muscle chaperones, with specific focus on their regulation and interaction with the UPS and the roles of both in client protein homeostasis during sarcomere assembly and maintenance. An overview of the discussed factors can be found in Table 2.

## 2. The Chaperones

A chaperone is defined by its ability to bind and help fold client proteins and prevent the aggregation of proteins in the cytoplasm [7,9,56]. Co-chaperones are non-client proteins that assist chaperones in protein folding and stimulate the ATP cycling of molecular chaperones [57,58]. As assembly factors, chaperones also aid in the building of oligomers (e.g., sarcomere thin and thick filaments in muscle cells). Chaperones can be ubiquitously expressed or tissue specific (see Table 2) and have hundreds of client or target proteins as the steps involved in protein folding are generally the same (e.g., members of the heat shock protein family) [59,60]. Chaperones fold client proteins but their specificity for targets within tissues and stages of development is often determined by their co-chaperones; therefore, the concentration and localization of these factors become important in determining folding or degradation outcomes. As such, the gene regulatory pathways controlling chaperones and their co-chaperones are not yet fully elucidated in muscle but some work has been done with respect to the myosin chaperones Hsp90, Unc45b and Smyd1b [61].

**Figure 2:**
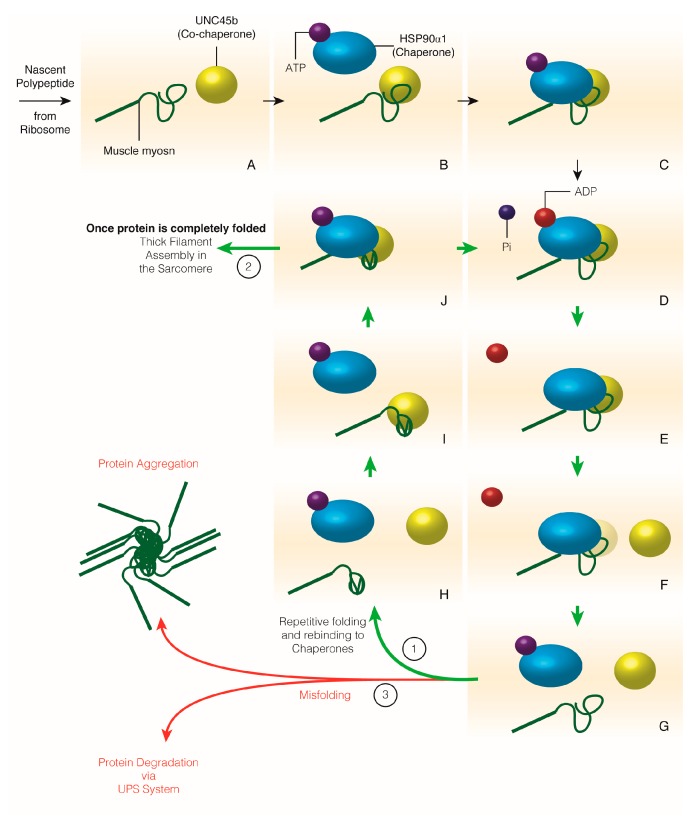
The folding of nascent client proteins by their chaperone and co-chaperones. Exemplifying the folding of the muscle myosin II globular head domain by Hsp90α1 and Unc45b as an example of molecular chaperones folding their client proteins. As proteins are synthesized by the ribosome, co-chaperones such as Unc45b can bind to the nascent polypeptide as it emerges from the ribosome (**A**,**B**). ATP-bound Hsp90α1 is recruited to nascent myosin polypeptides by Unc45b (**B**,**C**). Binding of Hsp90α1 causes a conformational change that hydrolyzes ATP to ADP that disassociates from Hsp90α1 (**C**–**E**). Unc45b releases Hsp90α1 and myosin, as Hsp90α1 rebinds cytoplasmic ATP, which allows the controlled folding of segments of unbound myosin (**F**–**H**). Hsp90α1 and Unc45b repeatedly rebind and fold myosin until it is completely folded (**I**,**J**; 1 & 2). If myosin is unable to be folded due to missing chaperones or incomplete binding, myosin (or any protein requiring folding) misfolds and can aggregate within the cell or be degraded via the ubiquitin proteasome system (3). Green arrows indicate the sequence of folding events that leads to a correctly folded protein to be incorporated into the sarcomere. Red arrows indicate the outcomes of misfolded proteins. Note that proteins can be discarded from any step of nascent protein folding and aggregate or be degraded.

The most well-known chaperone-client protein relationship is myosin heavy chains and their chaperones [58,62]. Through ATP hydrolysis (HSP protein family) and repeated binding and release of their client protein, these myosin chaperones allow controlled folding of myosin heavy chains (Figure 2).

## 3. Necessity of Chaperones for Sarcomere Assembly

**Figure 3:**
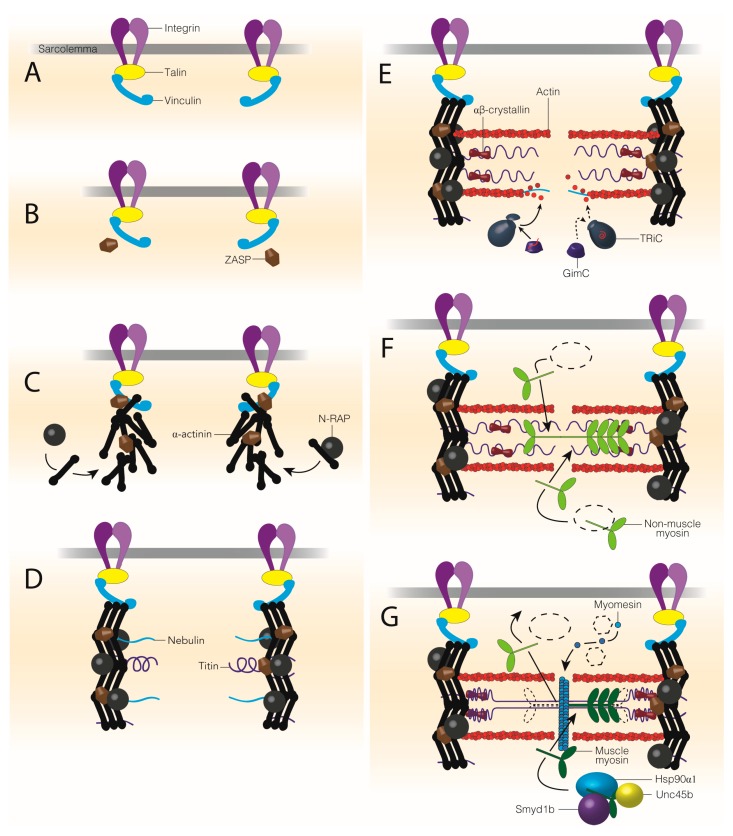
The Premyofibril Model of Sarcomere Assembly and Necessary Chaperones. Sarcomere assembly begins with the dimerization of integrins in the sarcolemma. Integrins recruit talin and viniculin to the sarcolemma to form protocostameres (**A**). ZASP localizes to the protocostameres to recruit α-actinin, which is folded and incorporated by its chaperone, N-RAP (**B**,**C**). The organization of α-actinin into Z-bodies likely recruits the protein giants nebulin and titin to the developing Z-discs (**D**). Nebulin and titin extend out from the Z-discs to the sarcomere center as Z-discs migrate away from one another to reach mature sarcomere length (**D**). GimC and TRiC fold actin before incorporating into thin filaments along the nebulin scaffold (**E**). Titin folding and integrity is maintained, in part, by αβ-crystallin during sarcomere assembly and muscle development (**E**). Non-muscle myosin II is proposed to aid in the alignment and formation of the thin filaments but the factors required for non-muscle myosin folding and incorporation are unknown ((**F**); dotted shape). In the final stages of sarcomere assembly, non-muscle myosin is replaced by muscle myosin II to form the thick filaments, which are assembled by Hsp90α1, Unc45b and Smyd1b (**G**). The M-line assembles either immediately after or simultaneously to thick filament formation and incorporates the tails of myosin heavy chains and the C-terminus of titin (**G**).

**Figure 4:**
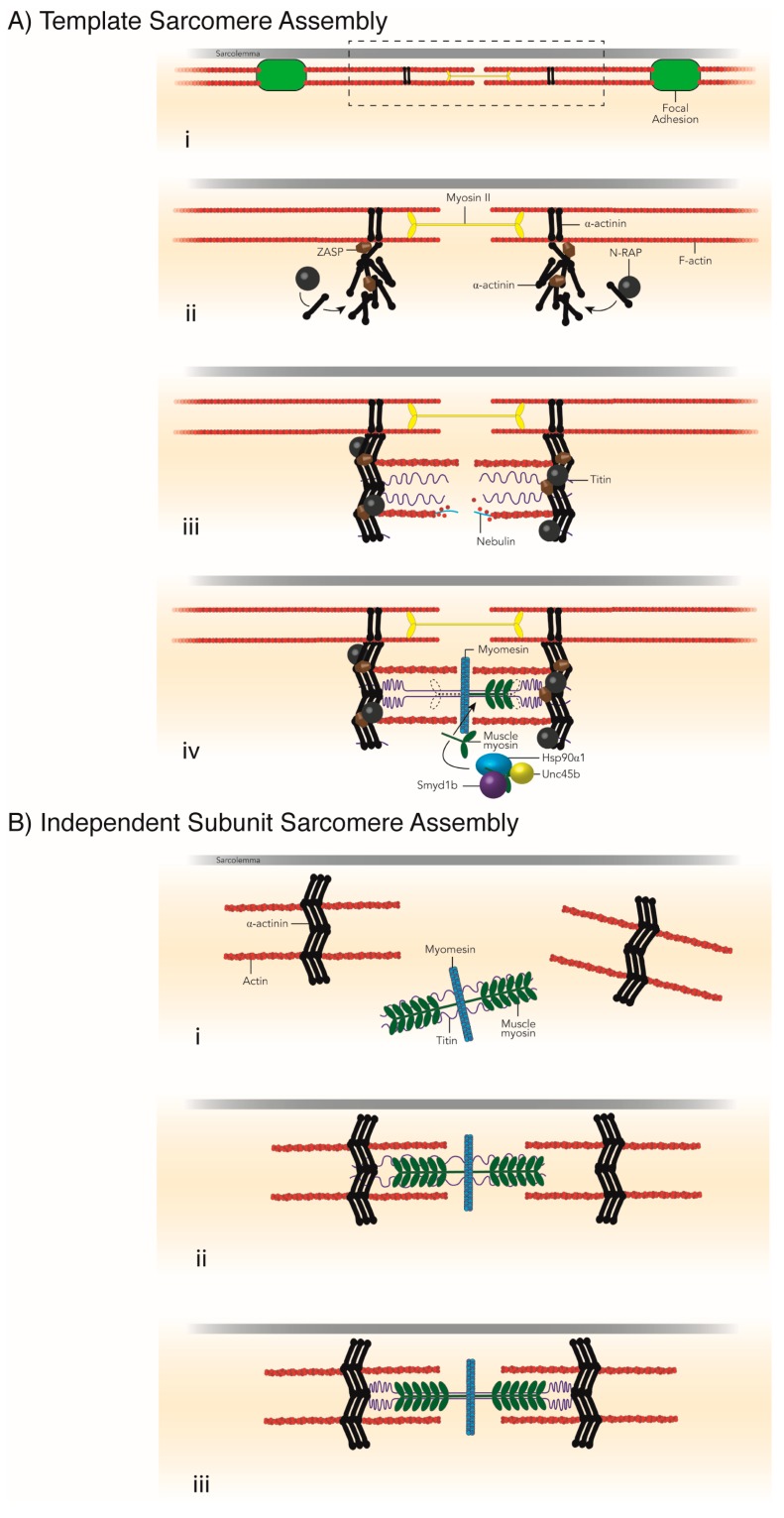
The Template and Independent Subunit Models of Sarcomere Assembly. The template model of sarcomere assembly suggests that sarcomeres require a template to form (**A**). The cell’s stress fibers, which have simple contractile structures, are proposed to be the templates for the formation of sarcomeres in myofibrils (**A**, **i**). Very similar to the premyofibril model, components of the Z-disc (such as α-actinin) form at stress fiber contractile sites (**A**, **ii**). Nebulin and titin extend from the Z-disc as actin is assembled into thin filaments along the nebulin scaffold (**A**, **iii**). Muscle myosin is folded and incorporated as thick filaments into the developing sarcomere by its chaperones and co-chaperones (**iv**). The independent subunit model for sarcomere assembly described sarcomere formation by the joining of pre-assembled subunits, or sections, of the sarcomere (**B**). These pre-assembled units consist of the Z-discs with attached actin thin filaments and the M-line combined with thick filaments and the protein giant, titin (**B**, **i**–**iii**). These units come together and physically connect at the sarcolemma to create the mature contractile sarcomere (**B**, **ii**,**iii**).

Of the various models of sarcomere assembly, the premyofibril model has the most supporting evidence both in vitro [47] and in vivo [63]. For an excellent review detailing these various models and the evidence for and against each, see [64]. In the premyofibril model of sarcomere assembly, α-actinin z-bodies and non-muscle myosin filaments form immature pre-sarcomeres, or premyofibrils (Figure 3) [64,65,66]. Incorporation of titin, muscle myosin and proteins of the M-line, mark the transition from premyofibrils to nascent and finally mature myofibrils as Z-discs, A-bands and M-lines are completed and the sarcomeres reach their final length and width. In addition to the evidence that supports the premyofibril model, this model is attractive as it is consistent with the idea that other factors are necessary for the growth and maturation of the sarcomere. This is in contrast to other models of sarcomere assembly, notably the Template [47,67,68,69] and Independent Subunit [47,69,70] sarcomere assembly models (Figure 4). Respectively, these models suggest sarcomere formation occurs along alpha-actinin, tropononin, tropomodulin and tropomyosin stress fiber templates [67] (Figure 4A), or form independently and are joined together along the length of titin [70,71] (Figure 4B). Neither, however account for the evidence that suggests that many sarcomere proteins themselves, such as myosin, a-actinin and the protein giant titin, cannot reach their native conformation without the assistance of chaperones. Furthermore, structures of the sarcomere require chaperones to help link components together, such as thin filament anchorage to the Z-disc or thick filament attachment to the M-line [72,73]. In this section, we will focus on the chaperones required for sarcomere protein folding and assembly of sarcomere structures.

### 3.1. Z-Disc Assembly

The assembly of the Z-disc occurs after the recruitment of talin, viniculin and ZASP to integrin at the muscle cell membrane [74,75,76,77,78]. Alpha-actinin localization requires ZASP and its organization into premature Z-discs, or Z-bodies, depends on N-RAP [35,79]. At present, no factor has been identified that is necessary for folding α-actinin. N-RAP binds α-actinin in the Z-disc and acts as a scaffold for thin filament assembly, acting as a bridge between Z-discs and thin filaments to help organize the I-Z-I structures of the sarcomere (Figure 3C–E) [72]. After the initial assembly of the sarcomere, desmin is incorporated into the Z-disc, by the molecular chaperone, αβ-crystallin, in order to attach sarcomeres of parallel myofibrils, anchor sarcomeres to the sarcolemma and maintain the structure and rigidity of the contractile unit [80,81].

### 3.2. Thin Filament Formation

Following Z-disc formation, the thin filaments begin to assemble along the nebulin scaffold that extends from the Z-disc toward the sarcomere center field [82,83]. The actin co-chaperone, GimC, binds actin as it is synthesized and prevents actin from aggregating in the cytoplasm. GimC then passes actin on to TriC, which completes actin folding and thin filament assembly (Figure 3E). Leiomodin 2 is important for elongating thin filaments to reach their mature length during sarcomere assembly [46]. Other chaperones, such as αβ-crystallin, associate with actin thin filaments but the function of this association is not well understood.

### 3.3. Non-Muscle Myosin Filaments

Non-muscle myosin II (NmmII) is incorporated into the developing myofibril between premature Z-discs simultaneously or immediately after Z-body formation and the beginning of thin filament assembly [47]. It has been suggested that NmmII aids in the alignment and assembly of actin thin filaments within the sarcomere but how NmmII integrates into the sarcomere is not understood [47,67,70,84,85]. Studies of NmmII are limited due to antibody specificity and the abundance of non-muscle myosins in every cell. Unc45b is a promising candidate as an NmmII chaperone as UNC45 in worms associates with NMM [86]. Non-muscle myosin II is replaced by muscle myosins, which may require an assembly chaperone or a member of the UPS system to target and remove NmmII from the sarcomere so that myosin thick filaments can be integrated into the sarcomere instead (Figure 3F,G).

### 3.4. Titin Folding and Incorporation

Titin, the largest known protein, stretches from Z-disc to the M-line and maintains sarcomere length. Due to its size, it is likely that titin has a host of assembly chaperones but the only chaperone required for titin filament assembly to date is αβ-crystallin [87,88]. Other chaperones, such as Hsp90α1, in addition to αβ-crystallin, localize to titin to help maintain titin integrity throughout sarcomere formation and muscle development [89,90]. It is likely that titin acts as a signaling hub of the sarcomere, with important roles in both sarcomere assembly and maintenance pathways, as well as serving as a platform for other chaperones involved in these roles [91].

### 3.5. Muscle Myosin Folding and Thick Filament Assembly

The abundant sarcomere protein, muscle myosin II has provided a target for elucidating the roles and coordination of molecular chaperones and the Ubiquitin Proteasome System. Many chaperones and co-chaperones are required to incorporate active myosin into the sarcomere [92,93,94,95,96]. Hsp40 is one of the first co-chaperones to bind myosin II and recruit Hsp70 [97,98]. Hsp90α1 and its co-chaperone, Unc45b, bind the hydrophobic amino acids of the myosin globular head domain and prevent it from misfolding [96,99,100]. Myosin II tails are capable of spontaneous dimerization but this dimerization appears to be timed for construction of the antiparallel thick filaments [99]. Once the myosin globular domain is folded, myosin heavy chains are organized into the hexamers of the thick filaments and anchored by the M-line (Figure 3G), replacing non-muscle myosins in the sarcomere. The HSP family are a major class of chaperones but they do not encompass all factors required for protein folding and assembly. For instance, Smyd1b (which has the protein structure of a histone methyltransferase and a myosin-binding domain) is also required for myosin thick filament assembly in the developing myofibril by making physical contacts with myosin, Unc45b and Hsp90α1 [73,95,101]. Whether Smyd1b is necessary for folding or incorporation, or both, has yet to be determined. Smyd1b may have a similar role to a co-chaperone as it binds skeletal muscle nascent polypeptide associated complex, or skNAC, which is found on ribosomes to bind newly synthesized proteins. It could be that skNAC recruits Smyd1b to myosin and this subsequently recruits Unc45b and Hsp90α1 respectively. The combination of these chaperones varies in the type of muscle tissue; Hsp90α1 is not required for cardiac myosin folding and Smyd1b is not required for slow myosin thick filament assembly [73,94,95,101,102,103].

### 3.6. M-Line Assembly

The assembly of the protein complexes that make up the M-line of the sarcomere is not well understood. No chaperones have yet been identified for 4 of the major components of the M-line: the C-terminus of titin, myomesin, obscurin and obscurin-like 1 [104,105,106,107]. The coiled tails of muscle myosins are part of the M-line acting as anchorage points for the thick filaments of the sarcomere. The C-terminus of titin recruits myomesin to the M-line and then localizes obscurin and obscurin-like 1 [105]. Similar to the localization of Z-disc components by assembly chaperones, the recruitment and incorporation of M-line proteins could be due to unidentified assembly chaperones. It is likely, based on the size and complexity of the M-line in comparison to other sarcomere structures/regions, that the M-line complexes require aid in protein folding and assembly.

This section covered the formation of the sarcomere with respect to chaperones and co-chaperones that are required for protein folding and assembly of the sarcomere. These assembly factors are necessary for the formation of the sarcomere and mutants of these factors result in various myopathies with many degrees of severity and onset [95,108,109]. It should be noted, that several myopathies result from mutations of sarcomere structural components and these mutations can have a wide effect on the function or stability of other sarcomere proteins (e.g., titin) [110].

## 4. Chaperone Homeostasis

The above sections discussed the overwhelming evidence that specific chaperones are required for proper sarcomere assembly and therefore survival of the organism. However, it is less intuitive but possibly equally important, that chaperone expression is regulated, as an overabundance of a particular chaperone can cause equally severe muscle defects. *C. elegans* UNC-45, zebrafish Unc45b and human skeletal muscle Unc45 (SM-Unc45) have been shown to cause muscle disorganization when overexpressed either transgenically or by blocking its degradation [15,111,112]. This suggests a model where chaperone homeostasis is a highly regulated process. In invertebrates, a mechanism for Unc45 removal has been elucidated by which Unc45, UFD2, CDC-48 and CHN-1 (*C. elegans* orthologue of CHIP [carboxyl terminus of Hsc70 interacting protein]) form a complex that is able to polyubiquitinate Unc45 [15,112]. The integrity of this system is important in humans as well, as mutations in p97 (human CDC-48), cause Inclusion Body Myopathy, which is associated with Paget disease of bone and Frontotemporal Dementia (IBMPFD). IBMPFD results in sarcomere assembly defects, which is attributed to excess SM-Unc45 [15]. While the mechanisms behind the sarcomere defects in Unc45/Unc45b/SM-Unc45 overexpression models are still being uncovered, some suggestions have been put forth. Willis and colleagues (2009) suggest that these defects result from too many Unc45 molecules attached to myosin, which (a) impedes sarcomere assembly and (b) causes the formation of aggregates [113]. Other models suggest that Unc45 overexpression may cause the sarcomere to assemble incorrectly, which is later recognized and disassembled [92].

Although Unc45 is known to work with Hsp90 and Smyd1b to fold myosin [95,101,114], no similar complexes have yet been reported for control of Hsp90 or Smyd1b turnover. As no data yet exists for Smyd1b overexpression, it is unsurprising that no complex regulating Smyd1b expression has yet been discovered. Importantly, the sarcomere defects associated with excess Unc45/Unc45b/SM-Unc45 also appear to proceed through an Hsp90 independent pathway [111]. While it does appear that Hsp90a/b isoform homeostasis is important for proper muscle specification [115], no consequences to sarcomere assembly have been described to date when Hsp90 is overexpressed in muscle [111]. This could be due to the general expression of Hsp90 such that it requires Unc45 cooperation in striated muscle, or it may indicate that the myosin chaperone actions of Hsp90 and Unc45 differ enough that overexpression of one is detrimental but the other is benign, such that only homeostasis of Unc45 is tightly regulated.

## 5. Chaperones beyond Assembly

The term “molecular chaperone” was first coined after observations that certain proteins required additional factors to facilitate proper binding and prevent precipitation [116]. The role of chaperones was later extended to include binding and folding client proteins after their synthesis, preventing the aggregation of misfolded proteins and aiding in the formation of macromolecular structures [117]. Recently, connections between chaperones and protein degradation have been established [55], linking chaperones with both autophagy and the UPS.

Chaperone-Mediated Autophagy (CMA) and Chaperone-Assisted Selective Autophagy (CASA) connect the chaperone system to protein quality control. CMA involves the delivery of a chaperone substrate across a lysosomal membrane in a ubiquitin-independent manner [118] and although Hsp70 is involved in this process [119], CMA has not been widely explored in skeletal muscle [42]. CASA involves the ubiquitin dependent degradation of a chaperone substrate by the lysosome [120]. Unlike CMA, there is strong evidence for CASA in skeletal muscle [42]. CASA was first described in the *Drosophila* Z-disc as a mechanism by which Starvin (Stv; *Drosophila* Bag-3) mediates the autophagic turnover of filamin in a CHIP ubiquitination dependent manner [120]. It is likely that this role of Bag-3 (Bcl-2-associated athanogene-3) is conserved in mammals as well, as Bag-3 homozygous null mice display normal Z-disc assembly, which deteriorates over time and results in eventual apoptosis [121]. Bag-3 also appears to be important in humans, with a severe muscular dystrophy linked to loss of Bag-3 [25]. Because Bag-3 is considered to be an Hsp70 (or ubiquitous Hsc70) co-chaperone [122,123] along with its many other proposed roles [123] the role of Hsp70 in muscle maintenance becomes an important and currently under-investigated question.

Evidence for chaperone and UPS collaboration comes from Chaperone-Assisted Proteasomal degradation (CAP), which describes the process by which a chaperone client is ubiquitinated and subsequently degraded by the proteasome [55,124,125]. Evidence for CAP comes from the Hsc/Hsp70 co-chaperone Bag-1, which associates with the 26S proteasome and has been proposed to physically link chaperone and proteasome systems [126]. Bag-1 has been shown to interact with CHIP [127], which provides a mechanism by which chaperone substrates could be ubiquitinated and targeted directly to the proteasome. Although CHIP is the only E3 ligase identified so far that binds to this complex, there are likely other E3 ligases involved in this role as CHIP knockout mice do not have severe muscle defects [128]. While it is very likely that this process is abundant in skeletal muscle, it has not been widely studied to date.

**Figure 5:**
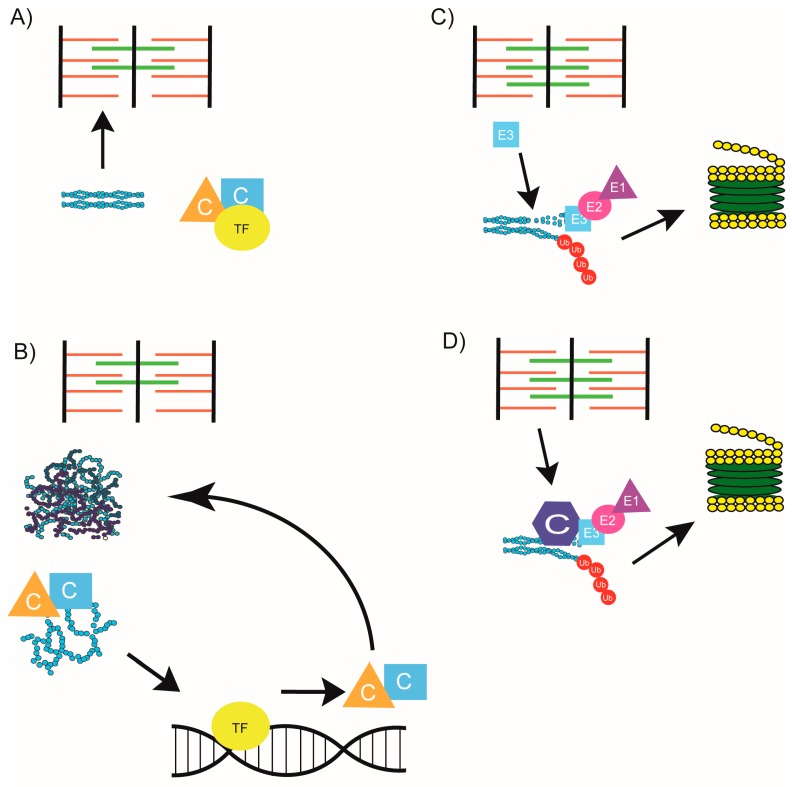
Damaged protein response in sarcomere assembly and maintenance. (**A**) In a healthy assembling sarcomere, chaperones (C) fold and incorporate muscle proteins; (**B**) when protein damage occurs during sarcomere assembly, chaperones dissociate from their complex to bind client proteins. Transcription factor (TF) translocates to the nucleus and initiates transcription of chaperones [61]. When protein damage occurs during sarcomere maintenance, either (**C**) a chaperone independent response occurs by which E3 ligases target damaged proteins and mark them for degradation or (**D**) a chaperone dependent response occurs by which chaperones and the UPS cooperate to target damaged proteins to the proteasome.

Together, the collaboration of chaperones with both autophagic and proteasome degradation systems suggests a hypothesis by which chaperones respond to their client protein when damaged regardless of when the damage occurs. As many chaperones remain associated with their client proteins or are localized on nearby sarcomere structures after sarcomere assembly, this hypothesis makes sense. However, recent evidence that myosin damage may occur without a subsequent upregulated response of myosin chaperones contradicts this model [61]. Differing from zebrafish with chaperone loss of function mutations—*steif* (Unc45b, [61] and *still heart* (smyd1b; [95], zebrafish with mutations outside the chaperone pathway but with similar muscle disorganization do not display an upregulation in myosin chaperones [61]. The zebrafish mutation, *sofa potato* (*sop*^fixe^), affects an acetylcholine receptor, which results in grossly normal muscle ultrastructure but smaller myofibrils and disorganized slow myosin [129]. *Ache* has a mutation in acetyl cholinesterase, which results in gradual disorganization of myofibrils and a loss of slow myosin over time [130]. Finally, *herzschlag* (*hel*) mutants carry a mutation somewhere between the I-band and A-band in the titin paralog *ttna* (*ttn2*; [131]). This mutation results in initially normal sarcomere assembly, which degenerates over time [131]. The absence of myosin chaperone upregulation in these mutants despite the myosin disorganization they display suggests that myosin chaperones are not directly responding to the disorganized protein itself [61]. Instead, it appears that Hsf-1 is responsible for inducing the myosin chaperone response [61]. However, it is still unclear why these chaperones do not react in *sop*, *ache*, or *hel* embryos. One difference between these zebrafish and those with mutations in myosin chaperones is that the initial folding and assembly of myosin into the sarcomere is unimpeded. The difference in myosin chaperone response is potentially due to the stage in which damage occurs, with myosin chaperones responding to initial folding and assembly defects through an Hsf-1 mediated response (Figure 5A,B). Should damage occur post assembly due to normal wear and tear, an unc45b/hsp90/smyd1b/hsf-1 independent response to myosin occurs. (Figure 5C,D) Whether this response is entirely chaperone independent, or involves a subset of muscle maintenance chaperones

## 6. Completing the Chain: Communication between Chaperones and the Proteasome

Although the misfolded protein response is still being uncovered, the above section discussed the increasing evidence that chaperones and the UPS cooperate throughout this process. Chaperones and the proteasome system are also linked by the discovery of the E3 ligase, CHIP, that can physically bind both Hsp70 [132] and Hsp90 [133] and is associated with the proteasome [133,134]. Furthermore, when Hsp90 is prevented from folding a client protein, the client protein is degraded by the proteasome [133]. Together, this strongly suggests that chaperones have roles beyond initial protein folding and link protein folding and protein degradation. Therefore, when a chaperone encounters a misfolded client protein, a decision must be made to either refold or degrade the protein. These decisions are referred to as protein triage decisions [135,136].

**Figure 6:**
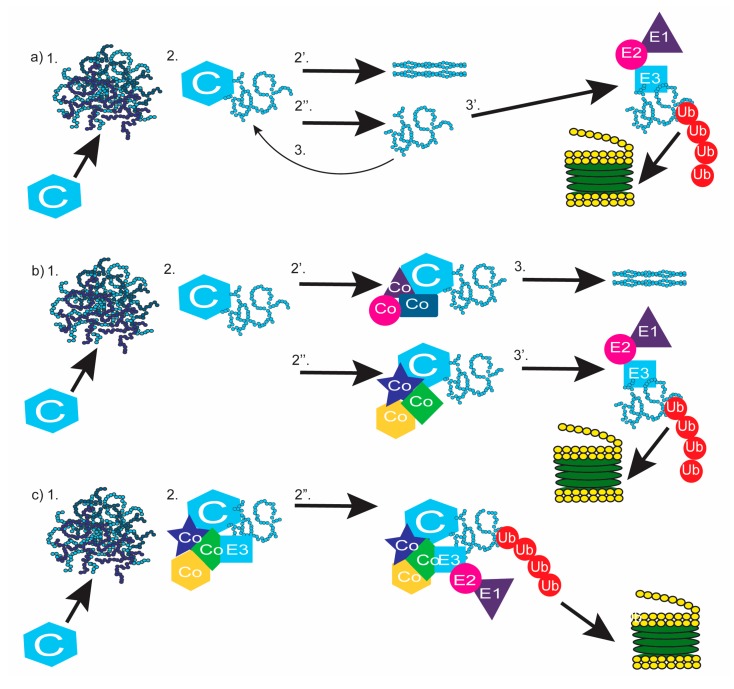
Models of chaperone and UPS cooperation in protein quality control. (**a**) Kinetic Model of Protein Triage. 1. Protein aggregate forms. Chaperones (C) are recruited. 2. Chaperones bind client protein and either succeeds in refolding (2′) or fail (2′′). 3. Misfolded proteins either rebinds chaperone or is targeted for degradation by the UPS; (**b**). cofactor mediated model of protein turnover. 1. Protein aggregate forms. Chaperones (C) are recruited. 2. Chaperone binds client protein and co-chaperones (co) are recruited and either promotes refolding of client protein (2′, 3. or degradation 2′′, 3′); (**c**) degradation complex model of protein triage. 1. Protein aggregate forms and Chaperones (C) are recruited. 2. Chaperone binds client protein and co-chaperones (co) are recruited, transforming the chaperone complex into the E3 ligase complex. 3. E3 complex is targeted to the proteasome for degradation.

How does the UPS recognize misfolded proteins? Many models have been presented [19] ranging from chaperones and the UPS existing as separate, competing, entities [135,136], to their coordination such that E3 enzymes deliver chaperone complexes and their ubiquitinated substrate, to the proteasome [124] (Figure 6). The simplest model of protein turnover is the “kinetic model of protein triage” which describes chaperones and the UPS as competing entities [135,136] (Figure 6A). In this model, chaperones respond to their misfolded client protein, likely recognizing exposed hydrophobic domains and attempt to refold it to its proper conformation. A misfolded protein that is not successfully refolded may be bound again by its chaperone, or by an E3 enzyme that also recognizes exposed hydrophobic domains [19]. In this model, whether a chaperone or an E3 enzyme binds the misfolded protein, is random [135,136], however concentrations of chaperones or E3 enzymes and damage to the client protein, would play an important role in which outcome is favored. If the misfolded protein is bound by its chaperone, the attempt to refolded cycle continues. If an E3 enzyme binds the misfolded protein, it is targeted for degradation.

An alternate model of protein turnover suggests that when a chaperone binds a misfolded client protein, it is the recruitment of specific co-chaperones that determines whether the client protein will be refolded or degraded [19,55]; (Figure 6B). Evidence for this model is abundant, mainly centers on the chaperones Hsp70 and Hsp90 and their substrates [19,55]. Co-factors Hip and Hop binding Hsp90 or Hsp70 has been shown to promote substrate folding [20,133,137], while binding of CHIP or Bag-1 promote substrate degradation [126,133,138,139] (For a schematic of Hsp/Hsc70 and Hsp90 associated co-chaperones and the refold/degradation consequence of the substrate, [55]). A lingering question remains as to how the appropriate co-chaperones are recruited. Like the previous model, refolding or degradation of the client protein likely depends on the initial damage to the protein as well as the presence and concentrations of its chaperone and co-chaperones. With all components being equal, this process will be stochastic [135]. In support of this, the addition of CHIP to lysates containing HOP and Hsp90 significantly decreases the number of HOP/Hsp90 complexes [133], suggesting that HOP can be outcompeted by CHIP. Furthermore, CHIP and HOP appear to bind the same location of Hsp70/Hsp90 [133] as do Bag-1 and HIP, which sterically hinder the binding of its competitor [55,140].

A third model of protein turnover suggests that the binding of a co-factor such as CHIP results in the transformation of a chaperone/client complex into a degradation complex, which is then transported to the proteasome [19,124,139]. Rather than CHIP and Bag-1 being thought of as co-factors in this model, these proteins and the chaperones they bind form a complex that is thought of as one big E3 ligase [124]. Substrate specificity will come from the chaperone’s affinity for their client [49], CHIP ubiquitinates proteins in the complex and then the complex is transported to and docked on the 26S proteasome by Bag-1 [126]; Figure 6C. This model differs from the “cofactor” model by the conceptualization of the entire complex as an active E3 ligase. Although these models are presented separately, they do not have to be mutually exclusive and it is feasible that certain co-factors promote folding, while others promote the transformation of the chaperone/substrate complex into a degradation complex. The binding and releasing of the folding co-factors or the degradation complex would be subject to competition, as outlined in the “kinetic model” making the best model of protein triage likely a combination of all three.

Much of the work done to formulate the models described above have been done in yeast, bacteria, or in vitro. Although these models have not been extensively studied in striated muscle, or in organisms containing striated muscle, it is possible that they could apply to this tissue as well. Further examination into these pathways in striated muscle could elucidate previously ignored treatments for myopathies listed in Table 1 and are therefore worth exploring. However, due to the tightly organized structure of the sarcomere, it is likely that an additional component in muscle is required to sense and report sarcomere damage. The protein titin, which stretches one-half sarcomere from Z-disc to M-line has been associated with sarcomere monitoring functions [91,141] and is a good candidate for regulating proteostasis. Links between titin and protein turnover have been established with the C terminal titin- kinase domain shown to be a binding site of the muscle specific ubiquitin ligases, MuRF1 [142], MuRF2 [143], as well as the protease Calpain 3 [26,144,145]. One role of this titin-kinase domain is to regulate gene expression in a contraction dependent manner, with MuRF2 and SRF translocating to the nucleus during an absence of contractions and regulating expression of genes such as myomesin [143]. However, as this domain also behaves as a reservoir for proteins involved in muscle maintenance, there is an obvious connection between titin and protein turnover that has yet to be fully elucidated.

MuRF2 belongs to the RING finger family of ubiquitin ligases and is closely related to MuRF1 [146], which has been shown to ubiquitin skeletal muscle myosin [147,148]. MuRF1 knockout mice are resistance to muscle atrophy [149], which is attributed to its RING domain [150] but are otherwise healthy and indistinguishable from wildtype animals [151]. Likewise, mouse knockout models of a third member of the MuRF family, MuRF3 do not display any abnormalities from wildtype animals, unless subjected to myocardial infarctions [148]. However, double-knockout (MuRF1^−/−^;MuRF3^−/−^) mice display a skeletal and cardiac muscle myopathy presenting similarly to human Myosin Storage Myopathies [148], which coupled with similar binding targets, [152] suggests that some functional redundancy exists between MuRF family members. As two members of this family are associated with titin [142,143], it is possible that titin regulates sarcomere protein turnover by docking ubiquitin ligases when muscle is healthy and releasing them when paralyzed [143]. This would constitute a chaperone independent response to sarcomeric damage, explaining the absence of myosin chaperone upregulation in zebrafish mutants with muscle damage [61]. However, it is impossible to eliminate the idea of a maintenance specific chaperone with this data alone and it is likely that both chaperone dependent and chaperone independent quality control systems exist together in striated muscle. We suspect that further research into the role of Hsp70 in muscle maintenance could fill in this gap. Likewise, “reporter” genes similar to titin could exist all over the sarcomere, including calsarcin at the Z-disc and myomesin and Mef2C at the M-line. How these systems work together and the signal transduction pathways regulating them will be an imperative focus of future muscle research.

## 7. Summary

Although the importance of protein turnover to muscle assembly and maintenance has become clear over the last few decades, the mechanisms and signaling pathways involved in this process still remain elusive. Both chaperone dependent and chaperone independent responses to protein damage have been identified but neither studied extensively in striated muscle. The onset and severity of the physiological and health consequences that occur when muscle proteostasis is interrupted (see Table 1), demands that understanding these processes are paramount to developing effective treatments and cures.

## Figures and Tables

**Table 1:** Sarcomere protein with assembly and maintenance factors and associated myopathy.

Sarcomere Protein or Structure	Chaperones Required for Assembly	Chaperones and UPS Members Required for Maintenance	Muscle Disease
fast myosin	Hsp40 Hsp70 Hsp90α1 Unc45b Smyd1b	CHIP MURF2	Inclusion Body Myopathy [17,18]
slow myosin	Hsp40 Hsp70 Hsp90α1 Unc45b	CHIP	Inclusion Body Myopathy [17,18,19,20]
actin	GimC TriC Leiomodin 2 Leiomodin 3	αβ-crystallin	Nemaline Myopathy [21,22,23,24]
α-actinin	ZASP N-RAP	MURF1 (associates with N-RAP in Yeast-2-hybrid)	Muscular Dystrophy [25] Nemaline Myopathy [24]
titin	αβ-crystallin	Hsp90α1 Smyd2 αβ-crystallin MURF1 (associates with titin in Yeast-2-hybrid) Calpain3	Limb Girdle Muscular Dystrophy [26,27,28] Tibial Muscular Dystrophy [29,30,31] Dilated Cardiomyopathy [32,33]
nebulin	Unknown	Unknown	Nemaline Myopathy
non-muscle myosin	Unc45 is a likely chaperone candidate	Unknown	Unknown
desmin	αβ-crystallin	Asb2β	Desmin Related Myopathies (DRM) Hypertrophic Cardiomyopathy [34]
troponin I	Unknown	MURF1	Nemaline Myopathy [21]

**Table 2:** Factors implicated in sarcomere assembly and maintenance.

Factor Name	Homologues Implicated in Muscle	Factor Type	Expression	Model Organism
UNC45	(SM-Unc45) Unc45b	Chaperone/Co-chaperone	Cardiac, fast and slow skeletal muscle	Human, Mouse, *Xenopus*, Zebrafish, *C. elegans*
	(GC-Unc45) Unc45a	Co-chaperone	Generally Expressed	Human, Mouse, Zebrafish
Hsp90	Hsp90α1	Chaperone	Cardiac, Skeletal muscle and Neural Tissue	Zebrafish
	Hsp90α2	Proposed chaperone	Cardiac, Skeletal muscle and Neural Tissue	Zebrafish
	Hsp90αβ	Unknown	Generally Expressed	Zebrafish
Smyd1	m-Bop/Smyd1b	Chaperone/Co-chaperone	Cardiac, Fast skeletal muscle	Mouse/Zebrafish
	Smyd1a	Proposed chaperone	Skeletal Muscle	Zebrafish
Smyd2	Smyd2a	Methyltransferase	Generally Expressed	Mouse, Zebrafish
αβ-crystallin	αβ-crystallin/CRYAB/Hspb1	Chaperone	Cardiac and Skeletal muscle	Human, Mouse, Zebrafish
MuRF1/Trim63	MuRF1/Trim63	E3 enzyme	Cardiac and Skeletal muscle	Mouse, Zebrafish (Zebrafish have Trim63a and Trim63b)
MuRF2/Trim55	MuRF2/Trim55	E3 enzyme	Cardiac and Skeletal muscle	Mouse, Zebrafish (Zebrafish have Trim55a and Trim55b)
MuRF3/Trim54	MuRF3/Trim54	E3 enzyme	Cardiac and Skeletal muscle	Mouse, Zebrafish
MuRF4/Trim101	MuRF4/Trim101	E3 enzyme	Skeletal Muscle	Zebrafish
Bag-1	Bag-1	Co-chaperone	Generally Expressed	Human, Mouse, Zebrafish
Bag-3/Starvin	Bag-3/Starvin	Co-chaperone	Striated muscle Z-disc	Human, Mouse, Zebrafish, *Drosophila*
Stub1/CHIP	Stub1/CHIP	Co-chaperone/E3 enzyme	Cytosol, ER	Human, Zebrafish, Mouse, *C. elegans*
Hsf-1	HSf-1	Transcription Factor	Generally Expressed	Humans, Mice, Zebrafish, *C. elegans*
Hsc-70	Hsp70	Chaperone	Generally Expressed Under Heat Shock	Human, Mice, Zebrafish, *E. coli*
	Hsp70-1	Chaperone	Generally Expressed Under Heat Shock	Human, Mouse, Zebrafish
	Hsp70-3	Chaperone	Unknown	Human, Mouse, Zebrafish
ST13/Hip	Hip	Co-chaperone	Cytoplasm	Human, Mouse, Zebrafish, *Drosophila*
STIP1/Hop	STIP1/Hop	Co-chaperone	Nucleus, Cytoplasm	Human, Mouse, Zebrafish, *C. elegans*, *Drosophila*
Calpain-3	Calpain-3	Protease	Generally Expressed	Human, Mouse, Zebrafish
SRF	SRF	Transcription factor	Nucleus	Human, Mouse, Zebrafish
UFD2/Ube4b	UFD2/Ub24b	E3 enzyme	Nucleus, Cytoplasm	*C. elegans*, Human, Mouse, Zebrafish
CHN-1	CHN-1	GTPase activating protein	Cytosol	Human, *C. elegans*
p97/Vcp/CDC48	p97/CDC48	ATPase	Generally Expressed	Human, Mouse, Zebrafish, *C. elegans*
